# Rho Family GTPases in Cancer

**DOI:** 10.3390/cancers13061271

**Published:** 2021-03-12

**Authors:** Suranganie Dharmawardhane

**Affiliations:** Department of Biochemistry, School of Medicine, University of Puerto Rico Medical Sciences Campus, San Juan, PR 00936, USA; su.d@upr.edu

This Special Issue containing seminal contributions from international experts highlights the current understanding of Rho GTPases in cancer, with an emphasis on recognizing their central importance as critical targets for cancer therapy and for chemosensitization of current therapeutic strategies. A comprehensive review by Jung et al. [1] gives an overview of the dysregulated Rho GTPases in multiple cancers, discoursing on their modes of regulation and potential targeted therapeutic strategies. Dr. Nandini Dey’s group discusses the pivotal role of Rac1 in solid tumors, which contributes to therapy resistance [2]. This review describes not only the mechanisms by which Rac1 regulates the actin cytoskeleton and thus motile mechanisms leading to metastasis and epithelial to mesenchymal transition (EMT) but also their function in pro-proliferative and pro-survival signaling, which directly contributes to tumor growth. Rac1 is also featured in a review by Drs. Kotelevets and Chastre [3], which outlines how Rac1 acts as a critical regulator of intestinal differentiation, leading to metastatic colorectal cancer. This review focuses on a range of Rac1 signaling pathways that are specifically dysregulated, outlining their contribution at each step of colorectal cancer progression.

New regulatory mechanisms for Rho GTPases are presented in a review by Humphries et al. [4], who describe microRNAs as novel targets for Rho GTPase regulatory proteins in cancer. A unique paradigm for Rho GTPases is also espoused by Streit et al. [5], who describe how neuroendocrine secretion is regulated by Rho GTPases during vesicle trafficking.

Novel directions for Rho GTPase guanine nucleotide exchange factors (GEFs) are also highlighted in a number of peer-reviewed research articles. Using in silico analyses and in vitro experimental studies in keratinocytes, Lorenzo-Martin et al. [6] implicate the Rho GEF Vav2 in stem-cell-like gene expression in head and neck cancer and associate Vav2 expression with poor patient prognosis. Baker et al. [7], in an intriguing article, show that the Rac.GEF p-REX1 actually does not contribute to Rac1 activation in prostate cancer, as previously thought. Using established androgen-insensitive prostate cancer cell lines, the authors demonstrate that Rac.GTP activation is dependent on a novel mechanism that is sensitive to elevated calcium levels.

Two articles by Dr. Hendrick Ungefroren’s group contribute to the role of the constitutively active Rac1B splice variant in transforming growth factor (TGFβ) signaling [8,9]. The authors elucidate the mechanisms by which Rac1B acts to inhibit TGFβ-1-dependent cell migration. In one paper, they show that Rac1B from poorly differentiated mesenchymal cancer cell lines regulates SMAD7, which is an inhibitory SMAD in TGFβ signaling, thus suppressing TGFβ-induced cell migration. This decrease in TGFβ-induced migration and growth is further dissected in a second article, where the authors connect Rac1B regulation of proteinase-activated receptor-2 (PAR-2) to the downregulation of the TGFβ receptor ALK5 in contributing to the suppression of TGFβ signaling in Panc1 pancreatic cancer cells.

Finally, Dyberg et al. implicate the downstream effector of Rho, Rho kinase (ROCK), in EMT and medulloblastoma growth by demonstrating that ROCK mRNA is preferentially expressed in metastatic tumors [10]. They used the ROCK inhibitor RKI-1447 to show the utility of targeting ROCKs in neuronal cancers.

In summary, herein, you will find timely articles on the ubiquitous role of Rho GTPases in cancer and how understanding their mechanisms of action can lead to the design and development of targeted therapeutic strategies.
